# Anthocyanins and betalains in evolution: Why mutual exclusion remains the best‐supported model

**DOI:** 10.1111/plb.70254

**Published:** 2026-07-02

**Authors:** B. Pucker, M. I. Khan

**Affiliations:** ^1^ Plant Biotechnology and Bioinformatics, IZMB University of Bonn Bonn Germany; ^2^ Biochemistry and Molecular Biology Lab, Department of Biotechnology Gauhati University Guwahati Assam India

**Keywords:** ANS, arGST, DFR, DOD, evolution, phenylalanine, tyrosine

## Abstract

Anthocyanins and betalains are hydrophilic plant pigments with numerous physiological and ecological functions. The biosynthesis routes of anthocyanins and betalains differ, with anthocyanins being synthesized from phenylalanine via the general phenylpropanoid pathway, whereas betalains are derived from tyrosine. Although the precursors phenylalanine and tyrosine are present in all plants, there is no known plant in which both these pigments are co‐accumulated. Most plants synthesize anthocyanins, while certain families in the order Caryophyllales produce betalains. There is an apparent mutual exclusion of these two plant pigments. Over the past five decades, evidence has accumulated supporting this theory of mutual exclusion of the two pigments. However, recently published reports claim the presence of anthocyanins in well‐known betalain‐pigmented plants without providing the necessary evidence. Here, we critically evaluate the origins of such claims, show how methodological limitations and analytical misinterpretations have led to unsupported reports of anthocyanin–betalain co‐occurrence, and provide recommendations to ensure robust evidence standards in future studies of plant pigment biosynthesis.

## Graphical abstract

Anthocyanins and betalains are plant pigments produced through different biosynthetic pathways and are generally considered mutually exclusive. We examine claims of their co‐occurrence, arguing that methodological flaws and data misinterpretation have led to unsupported reports, and offer recommendations for more rigorous future research.

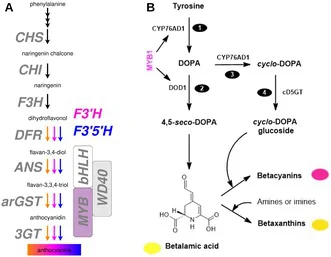

## BIOSYNTHETIC AND PHYLOGENETIC EVIDENCE OF MUTUAL PIGMENT EXCLUSION

**Fig. 1:**
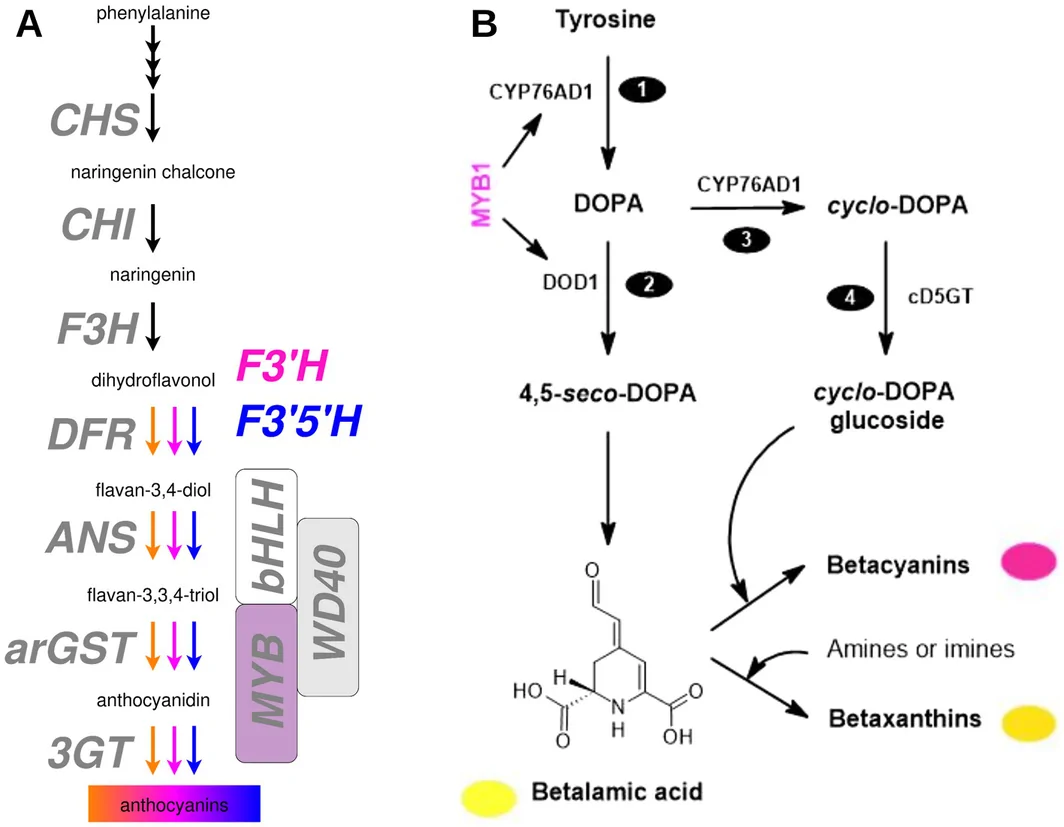
Simplified illustration of anthocyanin biosynthesis leading to pelargonidin (orange), cyanidin (pink), and delphinidin (blue) (A) and betalain biosynthesis based on knowledge about sugar beet (B). *CHS*, chalcone synthase; *CHI*, chalcone isomerase; *F3H*, flavanone 3‐hydroxylase; *F3*′*H*, flavonoid 3′‐hydroxylase; F3′5′H, flavonoid 3′,5′‐hydroxylase; *DFR*, dihydroflavonol 3‐reductase; *ANS*, anthocyanidin synthase; *arGST*, anthocyanin‐related glutathione S‐transferase; *3GT*, UDP‐dependent anthocyanidin 3‐O‐glucosyltransferase; *MYB*, Myeloblastosis; *bHLH*, basic helix–loop–helix; WD40, WD40 protein. The anthocyanin biosynthesis‐activating MBW complex is usually formed by a MYB of subgroup 6, a bHLH of subgroup IIIf, and an ortholog of the WD40 protein TRANSPARENT TESTA GLABRA 1 (TTG1). Numbered steps in the betalain biosynthesis are enzyme‐catalysed. Steps without any assigned numbers are spontaneous or pH‐dependent. The coloured ellipses indicate the corresponding colour of the compounds next to them. CYP76AD1, cytochrome P450 enzyme (multiple variants are known; CYP76AD1 is known to be involved in steps 1 and 2); DOPA, L‐3,4‐dihydroxyphenylalanine; DOD1, DOPA‐4,5‐dioxygenase (multiple variants are known). BvMYB1 from sugar beet has been shown to regulate the expression of *BvCYP76AD1* and *BvDOD1* (Hatlestad *et al*. 2015). cD5GT – *cyclo*‐DOPA‐5‐*O*‐glucosyltransferase. Betacyanins and betaxanthins are two subclasses of betalains.

Anthocyanins are hydrophilic plant pigments, well‐known for providing striking coloration of flowers and fruits, with numerous physiological and ecological functions, including protection against irradiation, protection against UV light, attraction of pollinators and seed dispersers, and camouflage (Grünig *et al*. 2025). The biosynthesis starts from phenylalanine via the general phenylpropanoid pathway and through a branch of the flavonoid biosynthesis (Fig. 1A). While the core biosynthesis appears largely conserved across plants, there are lineage‐specific differences in the decoration of anthocyanins with sugar moieties and other chemical groups (Grünig *et al*. 2025). Although anthocyanin pigmentation is widespread in plants, several families of the core Caryophyllales represent a notable exception. These lineages have lost anthocyanin pigmentation and synthesize yellow and red pigments of a different pigment class, betalains, which are not found in plants outside of the Caryophyllales (Timoneda *et al*. 2019). The exceptional pigmentation of many Caryophyllales species by betalains instead of anthocyanins was discovered about half a century ago and has been supported by numerous studies (Bate‐Smith 1962; Mabry 1964; Kimler *et al*. 1970; Ehrendorfer 1976; Stafford 1994; Clement & Mabry 1996; Tanaka *et al*. 2008; Brockington *et al*. 2011). Betalains are hydrophilic pigments and are involved in anthocyanin‐analogous functions in around 18 families in the core Caryophyllales (Timoneda *et al*. 2019). The betalain biosynthesis starts from tyrosine through hydroxylation by a CYP76AD1 enzyme to produce L‐3,4‐dihydroxyphenylalanine (L‐DOPA), which is converted by L‐DOPA‐4,5‐dioxygenase (DOD) to produce betalamic acid – the chromophore of all betalains (Fig. 1B) (Khan & Giridhar 2015). An analysis of the betalain‐biosynthesis‐specific enzyme DOD1 suggests that the betalain biosynthesis emerged at least four times independently within the Caryophyllales following a gene duplication of *LigB* which is also present in plants outside the Caryophyllales (Sheehan *et al*. 2020).

The anthocyanin loss in betalain‐pigmented taxa in the core Caryophyllales is fundamentally different from frequent pigmentation losses at the species level in land plants. However, the mutual pigment exclusion of anthocyanins and betalains could be explained by their functional redundancy (Timoneda *et al*. 2019). For example, only one of the two pigment types is required to achieve floral coloration and perform the ecophysiological functions, thus a loss of the other pigmentation biosynthetic pathway could happen through genetic drift or other drivers of evolution. Anthocyanin pigmentation was the ancestral state of the Caryophyllales and multiple independent losses of anthocyanin pigmentation resulted in the mutual exclusiveness of anthocyanins and betalains (Pucker *et al*. 2024). The anthocyanin biosynthetic genes in betalain‐pigmented lineages of the Caryophyllales are not activated due to mutations in the activating *MYB* gene (Pucker *et al*. 2024), which has been co‐opted for the betalain biosynthesis in the *Amaranthaceae* (Hatlestad *et al*. 2015). Additionally, the betalain‐pigmented lineages appear to lack a crucial anthocyanin biosynthesis enzyme, the anthocyanin‐related glutathione S‐transferase (arGST) (Pucker *et al*. 2024). Multiple independent transitions from phenylalanine‐based pigmentation with anthocyanins to tyrosine‐based pigmentation with betalains might be explained by the availability of both amino acids in the Caryophyllales, because a duplication of the arogenate dehydrogenase (ADH) gave rise to a feedback‐insensitive variant that could increase the availability of tyrosine at the expense of phenylalanine in the Caryophyllales (Lopez‐Nieves *et al*. 2018). In summary, these mechanisms can explain why the anthocyanin biosynthesis has been replaced by betalain biosynthesis in certain families of the Caryophyllales.

## QUESTIONABLE CLAIMS REGARDING THE PRESENCE OF ANTHOCYANINS IN WELL‐KNOWN BETALAIN‐PIGMENTED PLANTS

Despite strong evidence for betalain accumulation in well‐studied Caryophyllales species such as amaranth (*Amaranthus tricolor*), red beet (*Beta vulgaris*), quinoa (*Chenopodium quinoa*), purslane (*Portulaca oleracea*), and dragon fruit (*Hylocereus polyrhizus*/*Selenicereus undatus*), several research papers contain claims that anthocyanins are present in these plants (Table S1). Support for the presence of anthocyanins in betalain‐pigmented lineages is often drawn from controversial or retracted publications. For example, reports claiming the presence of anthocyanins in betalain‐pigmented pitayas have been criticized (Pucker *et al*. 2021; Khan 2022; Pucker & Brockington 2022) and have been retracted or are undergoing retraction but are still frequently cited. In the following, we examine recurring flaws in studies reporting anthocyanins in betalain‐pigmented species and discuss how these findings have been propagated through the literature despite substantial evidential weaknesses.

1. A recurring issue in the studies listed in Table S1 is the use of inappropriate analytical approaches or the lack of thorough methods. Spectrophotometric quantification without further confirmation using HPLC‐MS/MS following the guidelines laid down by the Metabolomics Standards Initiative (Sumner *et al*. 2007) is a frequent issue (Table S1). Authentication of the pigment identity is required because anthocyanins and betalains absorb light around 530 ± 10 nm, and both pigments are hydrophilic and show colour at pH 5–6 or below pH 4 (Stintzing & Carle 2004). A simple approach to differentiate the two pigments is based on the knowledge that anthocyanin extracts turn blue in alkaline pH, whereas betalain extracts are yellow/brown at the same pH. This can be easily carried out by adding a small amount of NaOH to the extract. Further, when the pH is made acidic, anthocyanin extracts regain the original extract colour instantaneously, whereas betalain extracts do not do so immediately. Given that betalains would be an adequate explanation for the coloration of the plant species listed in Table S1, claims regarding the presence of anthocyanins in these betalain‐pigmented taxa remain unsubstantiated and are inconsistent with the compelling evidence in the scientific literature.
2. Misinterpretation of transcriptomic data is a frequent issue. For example, a truncated transcript of an anthocyanidin synthase is highly abundant in the betalain‐pigmented *Mirabilis jalapa* although it does not result in a functional enzyme (Polturak *et al*. 2018). Some genes of the anthocyanin biosynthesis are still present and even expressed in betalain‐pigmented lineages, but with at least one gene missing, the anthocyanin biosynthesis is blocked (Pucker *et al*. 2024). The detection of transcripts of genes from the wider flavonoid biosynthesis is often mistaken for evidence of an active anthocyanin biosynthesis because the activity of these genes in other pathways is ignored.
3. The questionable studies were published between 2009 and 2026 in journals operating under peer‐review procedures. While these outlets are established, many are commercially operated journals rather than society‐backed publications, a distinction that may influence editorial priorities and peer‐review practices. The acceptance of claims that contradict the well‐documented mutual exclusivity of anthocyanins and betalains raises concerns regarding the consistency and subject‐specific rigour of the review process. In particular, it suggests that the evaluation may not have consistently involved reviewers and editors with specialized expertise in specialized plant metabolism and evolution.

## CONCLUDING REMARKS

### Mutual exclusion of anthocyanins and betalains is well‐established

Over the past 50 years, numerous biochemical and genetic studies have failed to provide convincing evidence that would contradict the theory of mutual exclusion of anthocyanins and betalain pigmentation. In summary, this can be seen as strong support for a well‐tested theory. It is also important to note that mechanistic explanations for the mutual exclusion, such as the presence or absence of crucial genes, further corroborate this theory. Therefore, strong evidence for the co‐occurrence of anthocyanin and betalain pigmentation in the same Caryophyllales species would be needed to disprove the mutual exclusion theory.

### Recommendations for future studies on anthocyanin/betalain pigmentation

Any study attempting to show the co‐occurrence of anthocyanins and betalains should present HPLC‐MS/MS analyses including reference compounds. The workflow needs to be described in a reproducible manner and all datasets have to be available in an established repository to enable independent validation. Given the high sensitivity of modern analytical instruments, it would be paramount to quantify the amount of detected compounds to demonstrate that they are not only traces, but can substantially contribute to pigmentation. Finally, such claims should be supported on the transcriptomic level by showing the presence of transcripts for all required steps (Fig. 1).

## AUTHOR CONTRIBUTIONS

BP and MIK conceptualized the review, prepared the original manuscript draft, and reviewed and edited the manuscript.

## Supporting information


**Table S1.** Some unsubstantiated claims of accumulation of anthocyanin in well‐known betalainic plants vis‐à‐vis substantiated claims.

## Data Availability

Data sharing not applicable to this article as no datasets were generated or analysed during the current study.
