# Supplementary material for: Anthocyanins and betalains in evolution: Why mutual exclusion remains the best‐supported model
Source: Plant Biol (Stuttg). 2026 Jul 2;28(6):1763–6. doi: 10.1111/plb.70254 (PMC13586639; doi:10.1111/plb.70254)
Supplement: Supplementary file 1 [file PLB-28-1763-s001.docx]

SUPPLEMENTARY MATERIAL

Anthocyanins and Betalains in Evolution: Why Mutual Exclusion Remains the Best-Supported Model

Boas Pucker^1,*^ Mohammad Imtiyaj Khan^2,*^

^1^ Plant Biotechnology and Bioinformatics, IZMB, University of Bonn, Kirschallee 1, 53115 Bonn, Germany

^2^ Biochemistry and Molecular Biology Lab, Department of Biotechnology, Gauhati University, Guwahati 781014. Assam, India

* Corresponding authors: pucker@uni-bonn.de, imtiyaj@gauhati.ac.in

**Table S1.** Some unsubstantiated claims of accumulation of anthocyanin in well-known betalainic plants^*^ vis-à-vis substantiated claims.

| Entry No. | Betalainic plant (Common name) | Family | Studies flagged | | | Confirmatory studies | | |
| --- | --- | --- | --- | --- | --- | --- | --- | --- |
|  |  |  | Unsubstantiated claim | Method of analysis | Reference | Substantiated claim | Method of analysis | Reference |
| 1 | *Amaranthus tricolor* L. (amaranth) | Amaranthaceae | Both anthocyanins and betalains are present | For anthocyanins: Spectrophotometer (absorbance at 530 nm);  For betalains: Spectrophotometer (absorbance at 540 and 475 nm) | (Wittayathanarattana et al., 2022a; Wittayathanarattana et al., 2022b) | Betalains are present | Spectrophotometer, high performance liquid chromatography (HPLC), and mass spectrometry (MS) | (Li et al., 2015; Chang et al., 2021; Kumari et al., 2023) |
| 2 | *Amaranthus tricolor* L.  (amaranth) | Amaranthaceae | Anthocyanins are present | Spectrophotometer (absorbance at 530 nm) | (Mila et al., 2024) |  |  |  |
| 3 | *Amaranthus* genotypes | Amaranthaceae | Anthocyanins are present | Full paper not accessible | (Yadav et al., 2013) |  |  |  |
| 4 | *Amaranthus cruentus* L.  (red amaranth) | Amaranthaceae | Anthocyanins are present | Spectrophotometer (absorbance at 510 nm; pH differential method) | (Paśko et al., 2009) |  |  |  |
| 5 | *Beta vulgaris* L. (red beetroot) | Amaranthaceae | Anthocyanins are present | Spectrophotometer (absorbance at 520 nm; pH differential method) | (Bilenler Koc et al., 2026) |  |  | (Nemzer et al., 2011) |
| 6 | *Beta vulgaris* L. (red beetroot) | Amaranthaceae | Anthocyanins are present | Spectrophotometer (absorbance at 520 nm) | (Guine et al., 2018) |  |  |  |
| 7 | *Chenopodium quinoa* Willd. (quinoa) | Chenopodiaceae | Anthocyanins are present | Spectrophotometer (absorbance at 510 nm; pH differential method) | (Paśko et al., 2009) |  |  | (Escribano et al., 2017; Imamura et al., 2025) |
| 8 | *Chenopodium quinoa* Willd. (quinoa) | Chenopodiaceae | Anthocyanins are present | Spectrophotometer (absorbance at 530 nm), untargeted metabolomics using LC-MS/MS | (Zhang et al., 2024) |  |  |  |
| 9 | *Chenopodium quinoa* Willd.  (quinoa) | Chenopodiaceae | Anthocyanins are present | Spectrophotometer (absorbance at 535 nm) | (Lata-Tenesaca et al., 2023) |  |  |  |
| 10 | *Chenopodium quinoa* Willd.  (quinoa) | Chenopodiaceae | Anthocyanins are present | Spectrophotometer (absorbance at 515 nm) | (Villacrés et al., 2022) |  |  |  |
| 11 | *Hylocereus polyrhiza* [(F. A. C. Weber) Britton and Rose] | Cactaceae | Anthocyanins are present | Spectrophotometer (absorbance at 535 nm) | (Guimarães et al., 2017) |  |  | (Wybraniec et al., 2001) |
| 12 | *Hylocereus polyrhiza* [(F. A. C. Weber) Britton and Rose]  (dragon fruit) | Cactaceae | Anthocyanins are present | Untargeted metabolomics using HPLC-QTOF | (Zhou et al., 2020) |  |  |  |
| 13 | *Hylocereus sp*. (red pulp) | Cactaceae | Anthocyanins are present | Spectrophotometer (absorbance at 538 nm) and HPLC monitored at 520 nm using authentic anthocyanin reference compounds | (Saenjum et al., 2021) |  |  |  |
| 14 | *Hylocereus polyrhiza* [(F. A. C. Weber) Britton and Rose]  (dragon fruit) | Cactaceae | Anthocyanins are present | Spectrophotometer (absorbance at 538 nm) | (Khoo et al., 2022) |  |  |  |
| 15 | *Hylocereus* spp. (red pulp) | Cactaceae | Anthocyanins are present | Spectrophotometer (absorbance at 538 nm) and HPLC monitored at 520 nm using authentic anthocyanin reference compounds | (Meng et al., 2026) |  |  |  |
| 16 | *Portulaca oleracea* L. (purslane) | Portulacaceae | Anthocyanins are present | Spectrophotometer (absorbance at 310 nm; pH differential method) | (Silva and Carvalho, 2014) |  |  | (Gandía-Herrero et al., 2009) |
| 17 | *Portulaca oleracea* L.  (purslane) | Portulacaceae | Anthocyanins are present | Untargeted metabolomics using UPLC-MS/MS | (Zhu et al., 2025) |  |  |  |

^*^ The evidence of these plants being betalainic taxa has been presented through rigorous phylogenetic analyses (Brockington et al., 2015; Sheehan et al., 2020). Also, the references to corresponding substantiated claims presented in the last column support the phylogeny.

**References**

**Bilenler Koc, T., Fırat, I., Karabulut, I., Boztepe, C., and Topalcengiz, Z.** (2026). Microencapsulation of beetroot anthocyanins: Investigation of degradation kinetics and modeling by using artificial neural networks. *ACS Omega* **11**:2270–2284.

**Brockington, S. F., Yang, Y., Gandia-Herrero, F., Covshoff, S., Hibberd, J. M., Sage, R. F., Wong, G. K. S., Moore, M. J., and Smith, S. A.** (2015). Lineage-specific gene radiations underlie the evolution of novel betalain pigmentation in Caryophyllales. *New Phytol.* **207**:1170–1180.

**Chang, Y.-C., Chiu, Y.-C., Tsao, N.-W., Chou, Y.-L., Tan, C.-M., Chiang, Y.-H., Liao, P.-C., Lee, Y.-C., Hsieh, L.-C., Wang, S.-Y., et al.** (2021). Elucidation of the core betalain biosynthesis pathway in Amaranthus tricolor. *Sci. Rep.* **11**:6086.

**Escribano, J., Cabanes, J., Jiménez-Atiénzar, M., Ibañez-Tremolada, M., Gómez-Pando, L. R., García-Carmona, F., and Gandía-Herrero, F.** (2017). Characterization of betalains, saponins and antioxidant power in differently colored quinoa (Chenopodium quinoa) varieties. *Food Chem.* **234**:285–294.

**Gandía-Herrero, F., Jiménez-Atiénzar, M., Cabanes, J., Escribano, J., and García-Carmona, F.** (2009). Fluorescence detection of tyrosinase activity on dopamine-betaxanthin purified from Portulaca oleracea (common purslane) flowers. *J. Agric. Food Chem.* **57**:2523–2528.

**Guimarães, D. de A. B., De Castro, D. dos S. B., Oliveira, F. L. de, Nogueira, E. M., Silva, M. A. M. da, and Teodoro, A. J.** (2017). Pitaya extracts induce growth inhibition and proapoptotic effects on human cell lines of breast cancer via downregulation of estrogen receptor gene expression. *Oxid. Med. Cell. Longev.* **2017**:7865073.

**Guine, R. P. F., Goncalves, F., Lerat, C., El Idrissi, T., Rodrigo, E., Correia, P. M. R., and Goncalves, J. C.** (2018). Extraction of phenolic compounds with antioxidant activity from beetroot (Beta vulgaris L.). *Curr. Nutr. Food Sci.* **14**:350–357.

**Imamura, T., Koga, H., Miyazato, A., Xu, Z., Shigehisa, R., Ohki, S., and Mori, M.** (2025). Identification of CqCYP76AD5v1, which is involved in betaxanthin biosynthesis in Chenopodium quinoa and its product, betaxanthin, inhibits amyloid-β aggregation. *Plant Biotechnol.* **42**:111–119.

**Khoo, H. E., He, X., Tang, Y., Li, Z., Li, C., Zeng, Y., Tang, J., and Sun, J.** (2022). Betacyanins and anthocyanins in pulp and peel of red pitaya (Hylocereus polyrhizus cv. Jindu), inhibition of oxidative stress, lipid reducing, and cytotoxic effects. *Front. Nutr.* **9**:894438.

**Kumari, M., Singh, H. B., and Khan, M. I.** (2023). Phenylalanine affects betalain biosynthesis and promotes ascorbic acid, α-tocopherol, and retinol accumulation in Amaranthus tricolor seedlings. *Acta Physiol. Plant.* **46**:17.

**Lata-Tenesaca, L. F., de Mello Prado, R., Ajila-Celi, G. E., da Silva, D. L., Junior, J. S. P., and Mattiuz, B.-H.** (2023). Iron biofortification in quinoa: Effect of iron application methods on nutritional quality, anti-nutrient composition, and grain productivity. *Food Chem.* **404**:134573.

**Li, H., Deng, Z., Liu, R., Zhu, H., Draves, J., Marcone, M., Sun, Y., and Tsao, R.** (2015). Characterization of phenolics, betacyanins and antioxidant activities of the seed, leaf, sprout, flower and stalk extracts of three Amaranthus species. *J. Food Comp. Anal.* **37**:75–81.

**Mila, K. J., Hassan, J., Hasan, Md. F., Alfagham, A. T., Ali, L., Islam, Md. S., Zubayer, Md., Gomasta, J., Ozaki, Y., Siddiqui, M. H., et al.** (2024). Nutritional composition, bioactive compounds and antioxidant potentiality of some indigenous vegetables consumed in Bangladesh. *Sci. Rep.* **14**:27699.

**Nemzer, B., Pietrzkowski, Z., Spórna, A., Stalica, P., Thresher, W., Michałowski, T., and Wybraniec, S.** (2011). Betalainic and nutritional profiles of pigment-enriched red beet root Beta vulgaris L. dried extracts. *Food Chem.* **127**:42–53.

**Paśko, P., Bartoń, H., Zagrodzki, P., Gorinstein, S., Fołta, M., and Zachwieja, Z.** (2009). Anthocyanins, total polyphenols and antioxidant activity in amaranth and quinoa seeds and sprouts during their growth. *Food Chem.* **115**:994–998.

**Sheehan, H., Feng, T., Walker-Hale, N., Lopez-Nieves, S., Pucker, B., Guo, R., Yim, W. C., Badgami, R., Timoneda, A., Zhao, L., et al.** (2020). Evolution of L-DOPA 4,5-dioxygenase activity allows for recurrent specialisation to betalain pigmentation in Caryophyllales. *New Phytol.* **227**:914–929.

**Silva, R., and Carvalho, I. S.** (2014). In vitro antioxidant activity, phenolic compounds and protective effect against DNA damage provided by leaves, stems and flowers of Portulaca oleracea (purslane). *Nat. Prod. Commun.* **9**:1934578X1400900115.

**Villacrés, E., Quelal, M., Galarza, S., Iza, D., and Silva, E.** (2022). Nutritional value and bioactive compounds of leaves and grains from quinoa (Chenopodium quinoa Willd.). *Plants* **11**:213.

**Wittayathanarattana, T., Wanichananan, P., Supaibulwatana, K., and Goto, E.** (2022a). A short-term cooling of root-zone temperature increases bioactive compounds in baby leaf Amaranthus tricolor L. *Front. Plant Sci.* **13**:944716.

**Wittayathanarattana, T., Wanichananan, P., Supaibulwatana, K., and Goto, E.** (2022b). Enhancement of bioactive compounds in baby leaf Amaranthus tricolor L. using short-term application of UV-B irradiation. *Plant Physiol. Biochem.* Advance Access published April 25, 2022, doi:10.1016/j.plaphy.2022.04.003.

**Wybraniec, S., Platzner, I., Geresh, S., Gottlieb, H. E., Haimberg, M., Mogilnitzki, M., and Mizrahi, Y.** (2001). Betacyanins from vine cactus Hylocereus polyrhizus. *Phytochemistry* **58**:1209–1212.

**Yadav, R. K., Joshi, S., Kumar, R., Varshney, R., and Jain, V.** (2013). Studies on diverse genotypes of amaranth (Amaranthus species) for their antioxidant and nutritional activities. *Indian J. Agric. Sci.* **83**:83–86.

**Zhang, M., Li, Y., Wang, J., Shang, S., Wang, H., Yang, X., Lu, C., Wang, M., Sun, X., Liu, X., et al.** (2024). Integrated transcriptomic and metabolomic analyses reveals anthocyanin biosynthesis in leaf coloration of quinoa (Chenopodium quinoa Willd.). *BMC Plant Biol.* **24**:203.

**Zhou, Z., Gao, H., Ming, J., Ding, Z., Lin, X., and Zhan, R.** (2020). Combined transcriptome and metabolome analysis of pitaya fruit unveiled the mechanisms underlying peel and pulp color formation. *BMC Genom.* **21**:734.

**Zhu, M., Wu, H., Fu, Y., You, L., Yang, C., Zhao, R., and Han, X.** (2025). Integrated metabolome and transcriptome analysis provides insights into anthocyanin biosynthesis in Portulaca oleracea. *BMC Plant Biol.* **25**:1774.
